# Early molecular changes predict cancer cachexia in LKB1‐deleted mouse models of NSCLC

**DOI:** 10.1002/ctm2.70360

**Published:** 2025-07-23

**Authors:** Gloriana Ndembe, Andrea David Re Cecconi, Federica Palo, Dorina Belotti, Laura Sala, Selena Foroni, Eugenio Scanziani, Rosanna Piccirillo, Massimo Broggini, Mirko Marabese

**Affiliations:** ^1^ Laboratory of Molecular Pharmacology, Experimental Oncology Department Istituto Di Ricerche Farmacologiche Mario Negri IRCCS Milan Italy; ^2^ Laboratory of Muscle Pathophysiology, Neuroscience Department Istituto Di Ricerche Farmacologiche Mario Negri IRCCS Milan Italy; ^3^ Laboratory of Tumor Microenvironment, Experimental Oncology Department Istituto Di Ricerche Farmacologiche Mario Negri IRCCS Milan Italy; ^4^ Department of Veterinary Medicine and Animal Sciences University of Milan Lodi Italy; ^5^ Mouse & Animal Pathology Lab Fondazione UniMi Milan Italy

1

Dear Editor

This preclinical study highlights the potential for early cachexia assessment in LKB1‐deficient non–small‐cell lung cancer (NSCLC) by molecular analysis of multiple tissues. In addition, it opens new avenues for investigating the role of IL‐12 as a protective factor against NSCLC‐related cachexia.

To investigate the role of LKB1 in cancer‐related cachexia, we used mouse cell lines derived from transgenic lung tumour nodules.^1^, ^2^ These cell lines, deleted in LKB1 but harbouring different Kirsten rat sarcoma virus (KRAS) mutations and the site of injection, were injected into immunocompetent mice. Regardless of the type of KRAS mutation, LKB1 deletion was associated with body weight loss (Figure S1).

To assess the possible role of sex,^3^ we injected KRAS^G12D^ (K) and KRAS^G12D^/LKB1^−/−^ (KL) NSCLC cells in male and female mice. K tumours grew faster than KL tumours in both sexes, with male mice facing faster overall tumour growth (Figures 1A and S2). Mice bearing KL tumour experienced rapid body weight loss, which was more pronounced in females. On day 26 post‐inoculation, KL mice had significantly lower body weights than K or control mice, whereas K mice had body weights similar to those of the control mice (Figure 1B,C).

**FIGURE 1:**
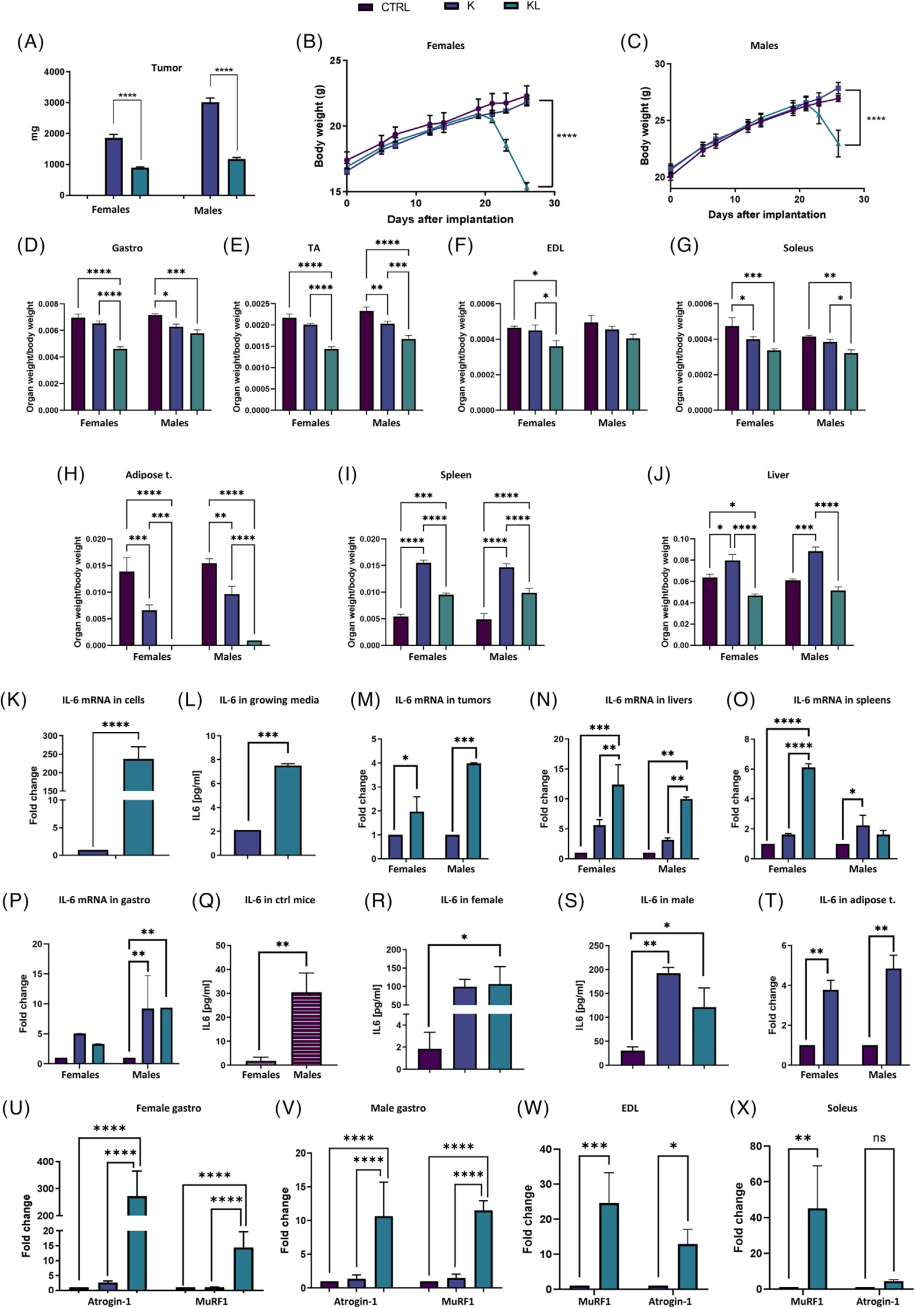
(A) Tumour weight and (B, C) body weight of female and male KL mice compared to K and control tumour‐free mice (CTRL). Weight of leg muscles (D–G), adipose tissue (H), spleen (I) and liver (J) of CTRL, K and KL mice. The organs were taken on the day of the sacrifice, 27th day after tumour implantation and the weights were normalised to initial body mass. (K) Level of IL‐6 gene expression measured in K and KL cultured cells by real‐time polymerase chain reaction (PCR). (L) Concentration of IL‐6 in the growing media of (K) measured by ELISA assay. IL‐6 gene expression assessed by real‐time PCR analysed in (M) tumour, (N) liver, (O) spleen and (P) muscle of K and KL tumour‐bearing mice intramuscularly injected. IL‐6 baseline gender differences (Q) and effect of K and KL tumour implantation assessed by ELISA assay in (R) female and (S) male mice. (T) IL‐6 gene expression analysed in the adipose tissue of male and female tumour‐free control mice and compared to K tumour‐bearing mice. Atrogin‐1 and MuRF1 gene expression in the gastrocnemius muscles (Gastro) of female (U) and male (V) mice, as well as in the extensor digitorum longus (EDL) (W) and soleus (X) muscles of female mice. Each experimental group included five mice. Statistical analysis was performed using two‐way analysis of variance (ANOVA; *p < .05, **p < .01, ***p < .001, ****p < .0001). Error bars indicate standard deviation (SD).

At sacrifice, KL mice showed statistically significant reductions in the gastrocnemius, tibialis anterior (TA) and soleus muscle weights. The extensor digitorum longus (EDL) muscle weight was significantly reduced only in females. Male K mice exhibited a significant reduction in gastrocnemius and TA muscle weights, while female K mice showed decreased soleus muscle weight (Figure 1D–G). Male KL mice also showed a severe reduction in visceral adipose tissue, which was completely absent in females. K tumours were associated with hepatosplenomegaly, whereas KL tumours were associated with liver mass reduction, especially in females (Figure 1H–J).

Histological analysis revealed hepatocellular atrophy, apoptosis, Kupffer cell hyperplasia and neutrophil infiltration in KL tumour‐bearing mice (Figure S3). Food consumption remained unchanged before weight loss, but KL females showed reduced consumption at later stages. K mice showed no differences in food intake (Figure S4). Immunocompromised KL‐bearing female mice also experienced weight loss, suggesting that the immune system is not the primary driver. In these animals also, K tumours grew faster than KL (Figure S5).

IL‐6, a key mediator of cachexia,^4^ was upregulated in KL cells compared to K cells in vitro (Figure 1K,L). In vivo, KL tumours showed higher IL‐6 expression than K tumours, especially in males (Figure 1M). Liver IL‐6 expression was elevated in both K and KL mice, with KL showing the highest levels (Figure 1N). IL‐6 expression in the spleen differed by sex: in females, only KL tumours bearing mice showed IL‐6 upregulation, whereas in males, K tumours bearing mice showed IL‐6 upregulation (Figure 1O). IL‐6 overexpression was detected in the gastrocnemius muscle of KL mice (Figure 1P).

Basal IL‐6 levels in control mice were higher in males than in females (Figure 1Q). Plasma IL‐6 levels were higher in K and KL tumours compared to controls in both sexes (Figure 1R,S).

Since adipose tissue is a primary source of IL‐6,^5^ its expression was analysed. Since KL mice lacked adipose tissue, only K‐bearing mice were compared to controls and showed strong IL‐6 upregulation (Figure 1T).

To determine the relationship between KL tumours and cachexia, we examined Atrogin‐1 and MuRF1, two muscle atrophy markers.^6^ Both were highly expressed in muscles from KL‐bearing mice (but not from K mice; Figure 1U–X).

To analyse the onset of cachexia, KL, K and control mice were monitored at five time points (7, 11, 17, 23 and 28 days post‐inoculation). Differences in tumour growth were observed starting from day 11, with K tumours growing faster than KL (Figure S6).

Body weight remained stable in all mice until day 23, while a decrease was found only in KL mice on day 28 (Figure 2A–E). Accordingly, tissue weight changes were detected only at the final time point, TP5, with KL mice showing absent visceral adipose tissue, whereas K‐bearing mice showed hepatomegaly and splenomegaly (Figure 2F–J).

**FIGURE 2:**
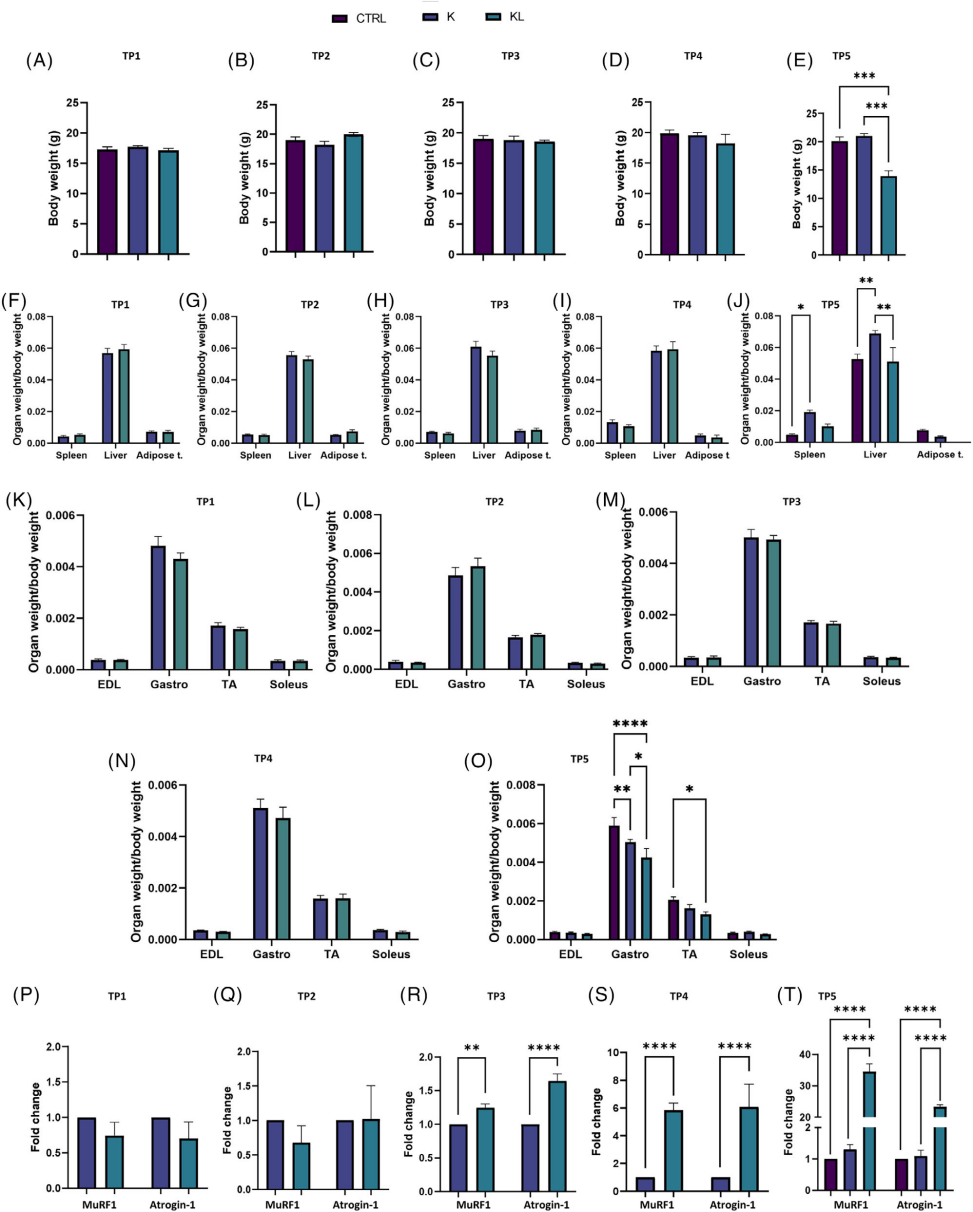
Body weight taken at 7 (A), 11 (B), 17 (C), 23 (D) and 28 (E) days after tumour cell injection. Spleen, liver and adipose tissue were collected and weighed at the same time points: 7 (A), 11 (B), 17 (C), 23 (D) and 28 (E) days after tumour cell injection. Gastrocnemius (Gastro), tibialis anterior (TA), extensor digitorum longus (EDL) and soleus muscles were collected and weighed at 7 (K), 11 (L), 17 (M), 23 (N) and 28 (O) days after tumour cell injection. Organs and muscles weights are normalised to initial body mass. Atrogin‐1 and MuRF1 gene expression in the gastrocnemius of female mice at 7 (P), 11 (Q), 17 (R), 23 (S) and 28 (T) days after tumour cell injection. For the analyses, the contralateral muscle to the tumour cell inoculation site was used. Each time point included five mice. Statistical analysis was performed using two‐way analysis of variance (ANOVA; *p < .05, **p < .01, ***p < .001, ****p < .0001). Error bars indicate standard deviation (SD).

Muscle weights remained unchanged until day 28, when KL mice showed reductions in gastrocnemius and TA muscle mass (Figure 2K–O). Food consumption analysis confirmed a decrease in KL mice at day 28 (not shown).

At the molecular level, we found a strong upregulation of Atrogin‐1 and MuRF1 in KL gastrocnemius muscle by day 17, without any weight loss (Figure 2P–T).

In adipose tissue, IL‐6 upregulation was found in KL mice by day 7 (Figure S7A). Furthermore, an increased UCP1 expression in KL adipose tissue was found on day 23, indicating white‐to‐brown fat conversion (Figure S7B).^7^ IL‐6 levels were elevated in KL spleens on day 11 and in livers on day 23 (Figure S7C,D).

To dissect the role of IL‐6, KL mice were treated with anti‐IL‐6 antibody. Treatment had no effect on either body weight, tumour size, muscle mass or Atrogin‐1/MuRF1 expression (Figure 3A–D). Although IL‐6 levels decreased in treated KL mice (Figure 3E), STAT3 activation was unaffected (Figure S8).

**FIGURE 3:**
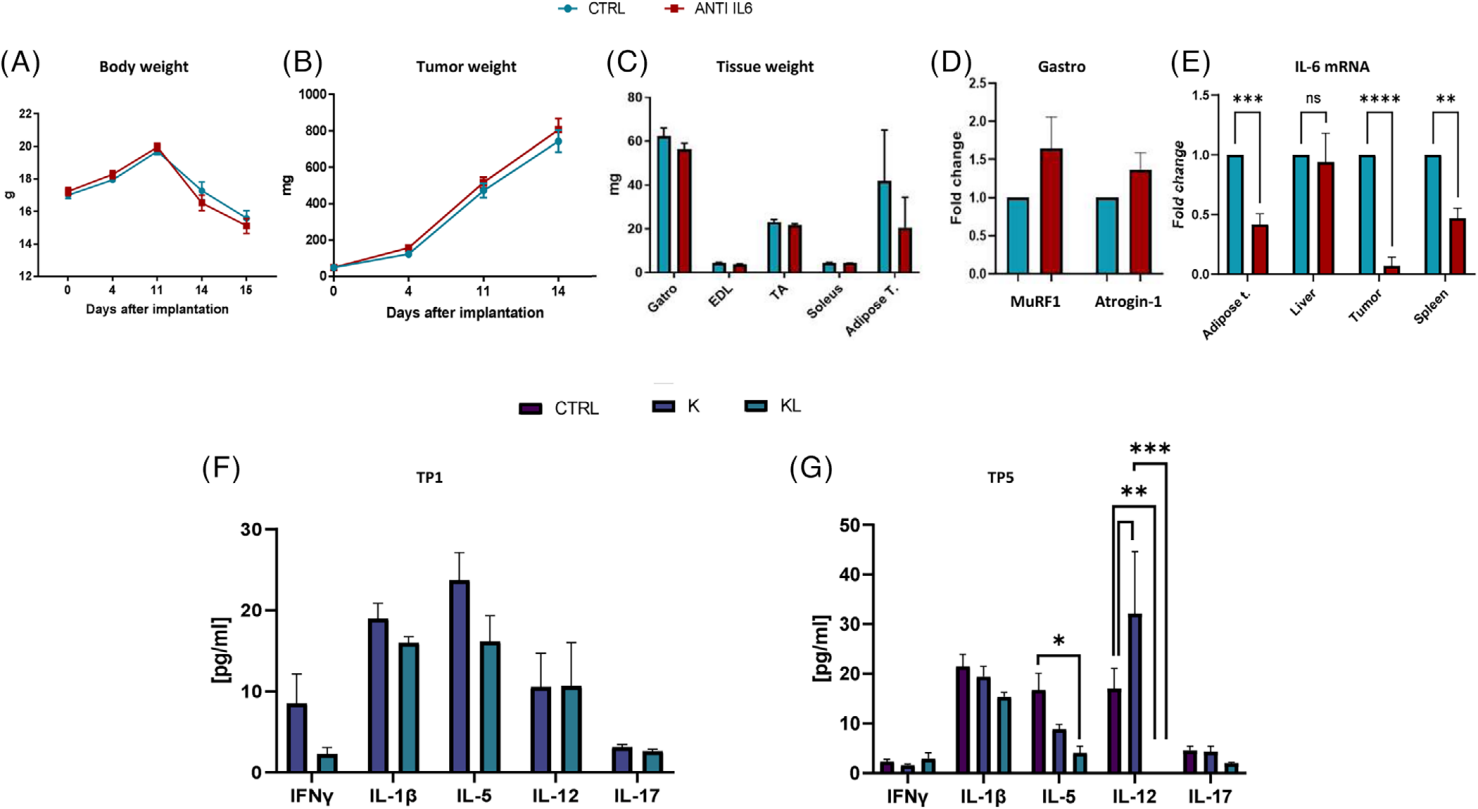
Effect of anti‐IL‐6 antibody on body weight (A), tumour growth (B), fat and lean mass (C). Atrogin‐1 and MuRF1 gene expression in the gastrocnemii of KL mice (D) and IL‐6 gene expression (E) in the adipose tissue, livers, tumours and spleen of KL mice were investigated. Each experimental group included five mice. A multiplex ELISA assay was utilised to measure the concentration of different cytokines involved in cancer‐related cachexia in the plasma of K and KL at 7 (F) and 28 (G) days after tumour cell injection. Statistical analysis was performed using two‐way analysis of variance (ANOVA; *p < .05, **p < .01, ***p < .001, ****p < .0001). Error bars indicate standard deviation (SD).

Multiplex ELISA analysis of cachexia‐related cytokines (IFNγ, TNFα, IL‐1β, IL‐5, IL‐12, IL‐13, IL‐17)^8^ at different time points showed no significant increase in KL plasma samples from KL host compared to K mice. Only IL‐12 levels were significantly reduced in KL mice at the last time point (Figure 3F,G). IL‐6 levels were not significantly different between K and KL mice at early stages, but increased at day 28 when compared to tumour‐free mice (Figure S9).

In conclusion, our results demonstrate that transcriptional changes in key tissues occur much earlier than the phenotypic manifestation of cachexia, with adipose tissue being the first to be affected by the presence of LKB1‐deficient tumours in preclinical NSCLC models. These data open the way to new potential interventions to prevent it. Among the cytokines analysed, although IL‐6 may play a role in the onset of cachexia, it is certainly not the only factor involved. We highlighted a potential protective role of IL‐12 that might provide further insights into the mechanisms underlying lung cancer‐associated cachexia.

## AUTHOR CONTRIBUTIONS

Conceptualisation: Gloriana Ndembe, Mirko Marabese, Massimo Broggini; Methodology: Gloriana Ndembe, Andrea David Re Cecconi, Massimo Broggini, Eugenio Scanziani, Rosanna Piccirillo; Investigation: Gloriana Ndembe, Andrea David Re Cecconi, Federica Palo, Dorina Belotti, Laura Sala, Selena Foroni; Funding acquisition: Massimo Broggini; Supervision: Mirko Marabese, Massimo Broggini; Writing, review & editing: all the authors.

## CONFLICT OF INTEREST STATEMENT

The authors declare no conflicts of interest.

## ETHICS STATEMENT

The IRFMN adheres to the principles set out in the following laws, regulations and policies governing the care and use of laboratory animals: Italian Governing Law (D.lgs 26/2014; Authorisation no. 19/2008‐A issued 6 March, 2008 by Ministry of Health); Mario Negri Institutional Regulations and Policies providing internal authorisation for persons conducting animal experiments (Quality Management System Certificate—UNI EN ISO 9001:2015 – Reg. No. 6121); the NIH Guide for the Care and Use of Laboratory Animals (2011 edition) and EU directives and guidelines (EEC Council Directive 2010/63/UE).

## Supporting information



Supporting Information

## Data Availability

Data generated or analysed during this study are included in this published article and its supporting information files. All the raw data are available upon request from the corresponding author.
