# Supplementary material for: Early molecular changes predict cancer cachexia in LKB1‐deleted mouse models of NSCLC
Source: Clin Transl Med. 2025 Jul 23;15(7):e70360. doi: 10.1002/ctm2.70360 (PMC12286895; doi:10.1002/ctm2.70360)
Supplement: Supplementary file 1 — Supporting Information [file CTM2-15-e70360-s001.docx]

**Early molecular changes predict cancer cachexia in LKB1-deleted mouse models of NSCLC.**

**Gloriana Ndembe^1#^, Andrea David Re Cecconi^2#^, Federica Palo^2^, Dorina Belotti^3^, Laura Sala^4,5^, Selena Foroni^1^, Eugenio Scanziani^4,5^, Rosanna Piccirillo^2^, Massimo Broggini^1*^, Mirko Marabese^1^**

^1^Laboratory of Molecular Pharmacology, Experimental Oncology Department, Istituto Di Ricerche Farmacologiche Mario Negri IRCCS, Milan, Italy.

^2^Laboratory of Muscle Pathophysiology, Neuroscience Department, Istituto Di Ricerche Farmacologiche Mario Negri IRCCS, Milan, Italy.

^3^Laboratory of Tumor Microenvironment, Experimental Oncology Department, Istituto Di Ricerche Farmacologiche Mario Negri IRCCS, Milan, Italy.

^4^Department of Veterinary Medicine, University of Milan, Milan, Italy.

^5^Mouse & Animal Pathology Lab, Fondazione Filarete, Milan, Italy.


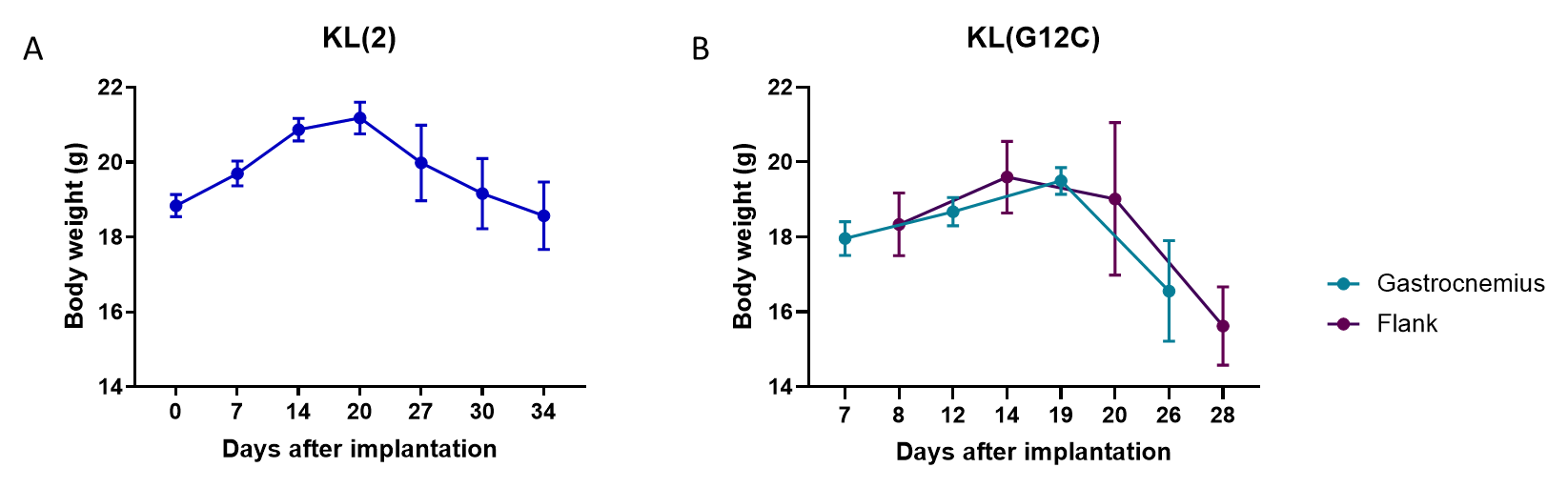


**Supplementary Figure S1. Body weight of female immunocompetent mice**: Immunocompetent mice were inoculated with (A) a second cell line KRAS^(G12D)^/LKB1^-/-^(KL2) in the gastrocnemius and (B) KRAS^(G12C)^/LKB1^-/-^ in the gastrocnemius or in the flank. Both cell lines deleted in *LKB1* induced body weight loss. Each experimental group included 5 mice. Error bars indicate SEM.

B

A

C

D

**Supplementary Figure S2. Tumor weight over time:** Tumor weight of (A) female and (B) male KL mice compared to K mice. K (C) and KL (D) tumor growth was compared in female and male immunocompetent mice. Each experimental group included 5 mice. Two-way ANOVA was used for statistical analysis (**p < 0.01, *** p < 0.001 ****p < 0.0001). Error bars indicate SEM.


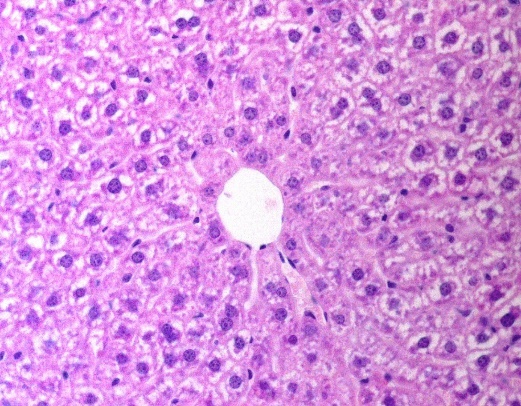


A


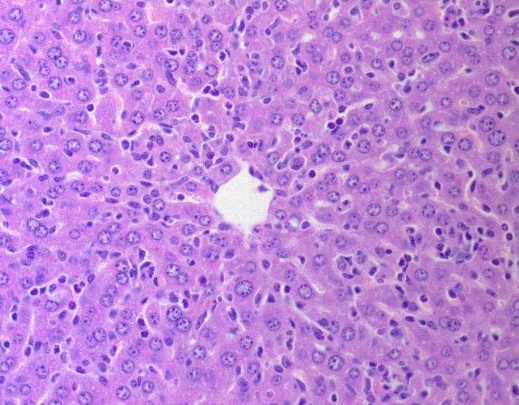


B

**Supplementary Figure S3. Liver histological evaluation:** Histological analysis of the liver obtained from control tumor-free mice (A) and KL mice (B). The samples collected at the end of the experiment reported in Figure 1 were formalin-fixed and trimmed according to (https://reni.item.fraunhofer.de/reni/trimming/) and section 1 and 2b were obtained. Five μm-thick tissue sections obtained from the paraffin blocks were stained with Hematoxylin and Eosin (H&E) and evaluated at light microscopy. Histopathology evaluation was made in a blind fashion, without knowledge of the treatment group.


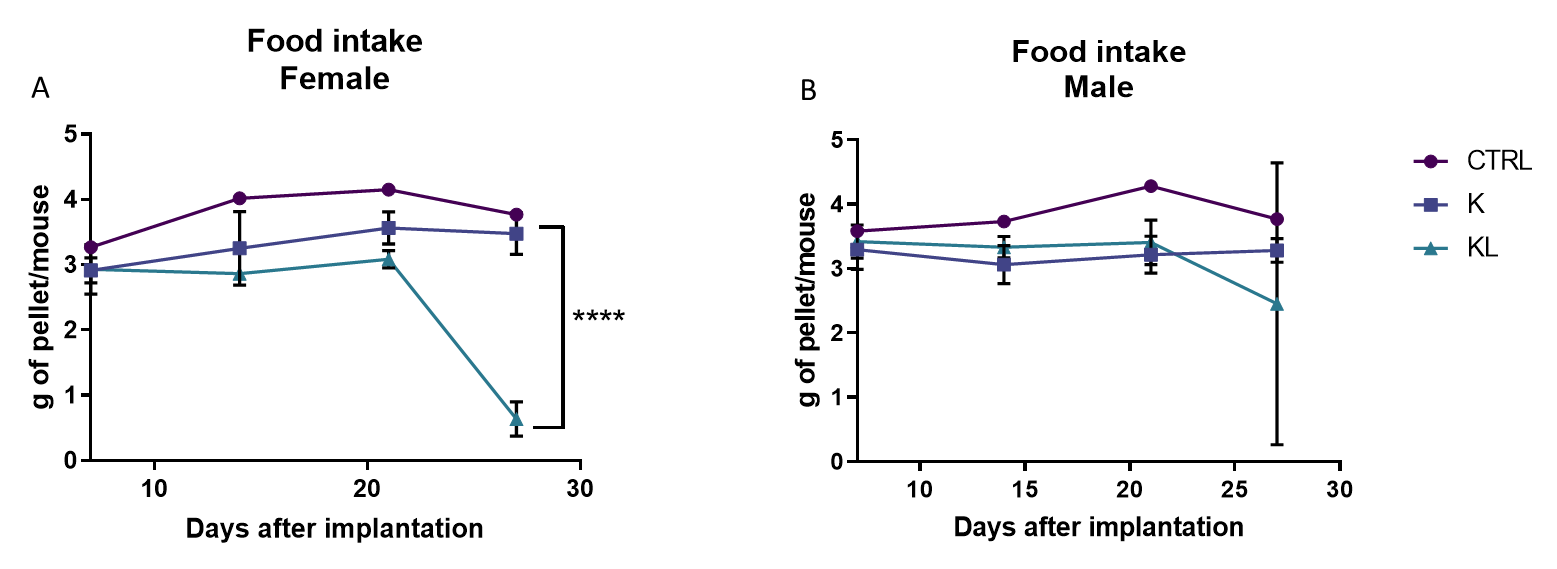


**Supplementary Figure S4. Food intake**: Evaluation of the grams of pellet consumed per mouse in female (A) and male (B) groups inoculated with K and KL cells, compared to control tumor-free mice. Each experimental group included 5 mice. Two-way ANOVA was used for statistical analysis (****p < 0.0001). Error bars indicate SD.


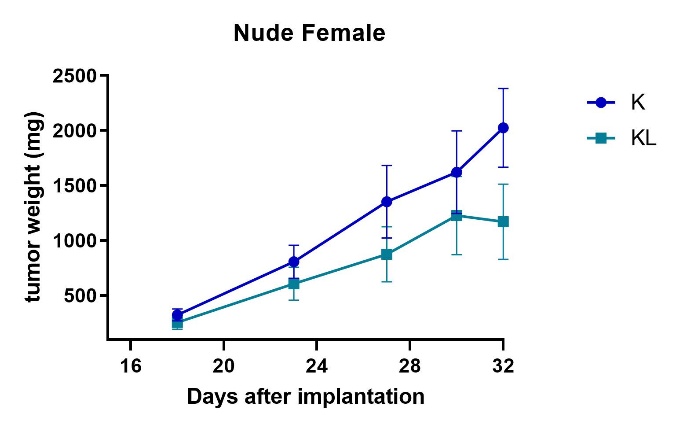


**Supplementary Figure S5 Body and tumor weight of nude female immunodeficient mice**: Graph of body weight (A) and tumor weight (B) of immunodeficient female mice intramuscularly inoculated with K and KL cell lines. Each experimental group included 5 mice. Two-way ANOVA was used for statistical analysis (**p < 0.01). Error bars indicate SEM.


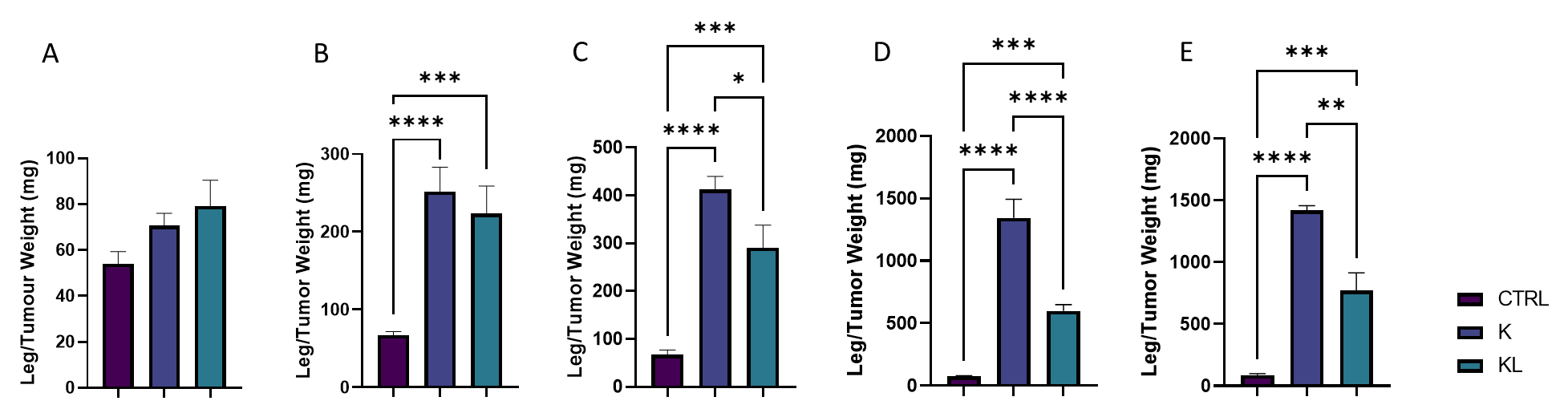


**Supplementary Figure S6. Tumor weight**: The measurements of tumor weight were taken at different time points: (A) 7, (B) 11, (C) 17, (D) 23, and (E) 28 days after tumor cell injection into the gastrocnemius. Tumor growth was compared to the leg weight of CTRL tumor-free mice. Each experimental group included 5 mice. Two-way ANOVA was used for statistical analysis (*p < 0.05, **p < 0.01, *** p < 0.001, ****p < 0.0001). Error bars indicate SEM.


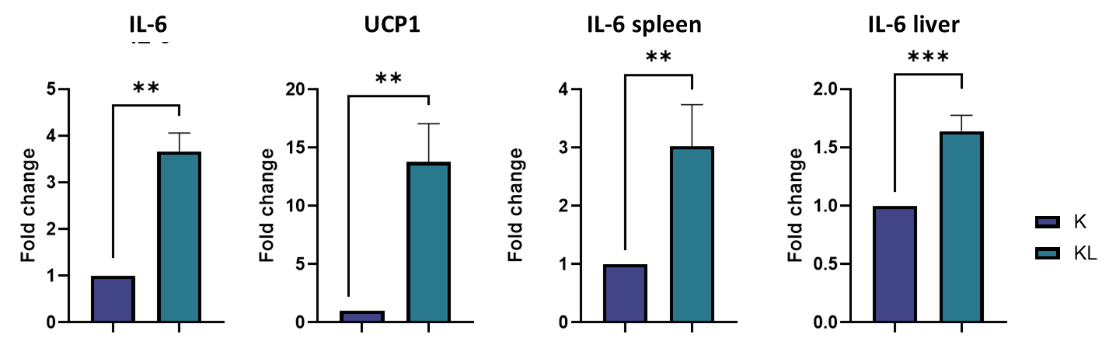


**Supplementary Figure S7. Molecular analysis of tissues:** Analysis of (A) IL-6 and (B) UCP1 gene expression in the adipose tissue of K and KL mice respectively 7 and 23 days after tumor implantation. IL-6 gene expression analysis in the (C) spleens of K and KL mice 11 days after tumor implantation and (D) livers of K and KL mice 23 days after tumor implantation. Each experimental group included 5 mice. T-test was used for statistical analysis (**p < 0.01, *** p < 0.001). Error bars indicate SD.


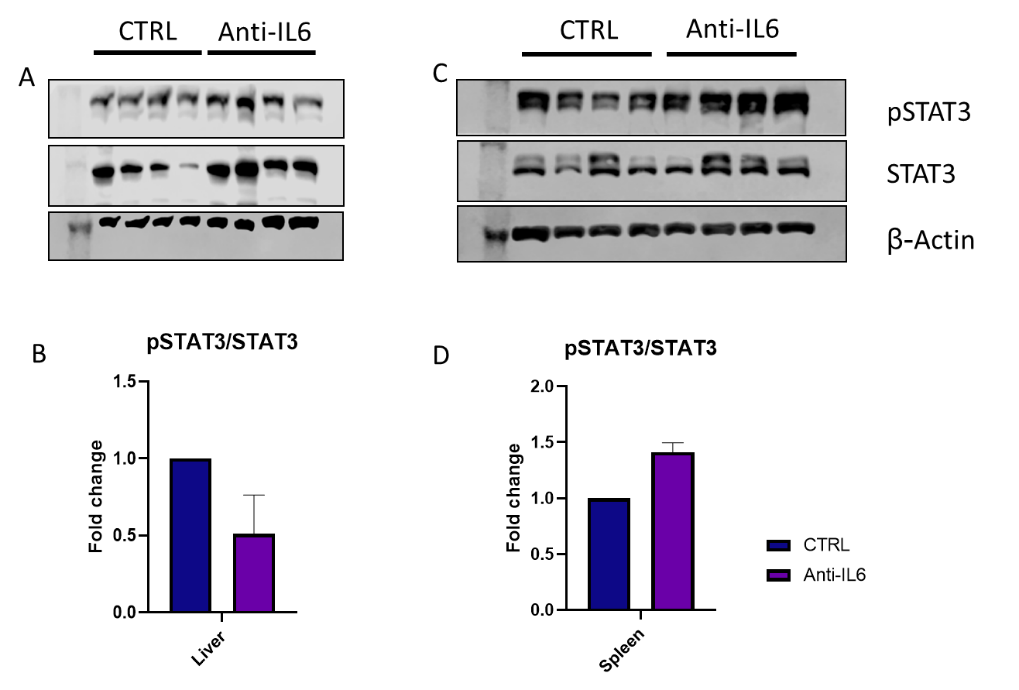


**Supplementary Figure S8. Analysis of STAT3 activation in tissues:** Representative immunoblot analysis of total and phosphorylated forms of STAT3 in the (A) livers and (C) spleens. Bar graphs (B for livers and D for spleens) show the quantification analysis of the active forms of STAT3 normalized to total STAT3. β-Actin was used as the loading control. The treated group with Anti-IL6 was compared to an untreated control group. Error bars indicate SD.


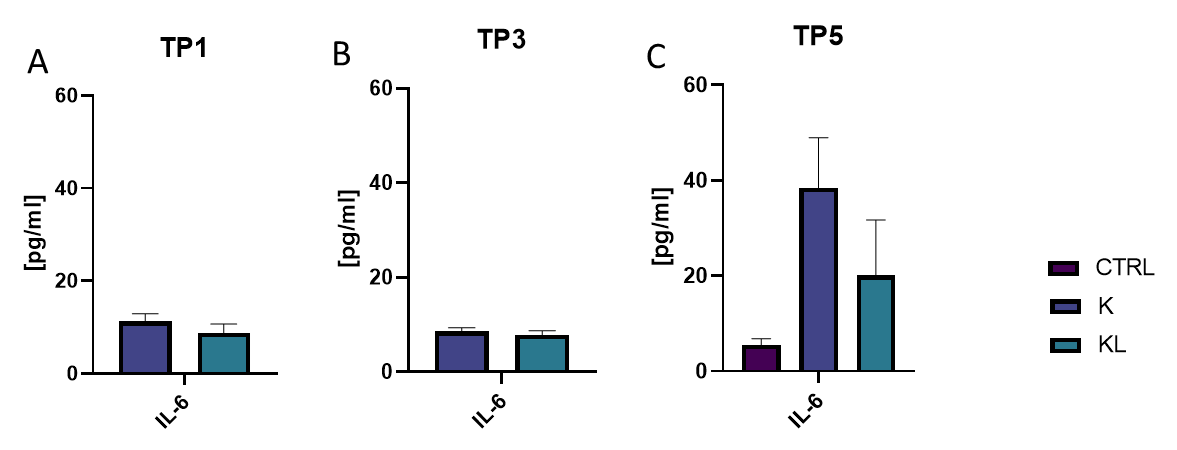


**Supplementary Figure S9. IL-6 plasma concentration:** To confirm the previous results we measured the concentration of IL-6 at (A) time point 1 (TP1, 7 days after tumor cell injection), (B) time point 2 (TP2, 11 days after tumor cell injection) and (C) time point 3 (TP3, 17 days after tumor cell injection). Two-way ANOVA was used for statistical analysis. Error bars indicate SD.

**MATERIALS AND METHODS**

*Cell lines***.**

Mouse tumor cell lines were generated from lung nodules of KRAS^G12D^/LKB1^wt^ (K) and *KRAS*^G12D^/*LKB1*^del^ (KL) transgenic mice as described in^1^, and cultured in Roswell Park Memorial Institute (RPMI)-1640 supplemented with 2mM of L-glutamine (Microgem) and 10% (v/v) fetal bovine serum (FBS, Euroclone).

The cell lines are routinely tested by polymerase chain reaction for mycoplasma contamination.

*Protein extraction and Western blotting.*

Proteins were extracted and visualized as reported in^2^. Immunoblotting was carried out with the following antibodies: anti-phospho-STAT3 (#9145), anti-STAT3 (#9139), purchased from Cell Signaling Technology. Anti-β-actin #sc-32268 was purchased from Santa Cruz Biotechnology. The secondary antibodies anti-rabbit #170-6515 and anti-mouse #170-6516 from BioRad were used.

*Mouse tumor models and in vivo treatments.*

For the evaluation of the induction of cancer-related cachexia, five-week-old male and female C57BL/6 mice and five-week-old female nude mice, purchased from Charles River Laboratories Italia S.p.a were used.

Mice were intramuscularly injected with K or KL cells in the gastrocnemius with 5x10^5^ or 10^5^ cells, respectively.

Food intake was measured once a week and was calculated by dividing the average number of grams of food consumed in each cage by the number of mice.

For the evaluation of the *in vivo* effect of IL-6 inhibition, five-week-old female C57BL/6 mice were injected in the gastrocnemius with 10^5^ K or KL cells and treated intraperitoneally (ip) twice a week with 200 µg of anti-IL-6 MP5-20F3 monoclonal antibody (BE0046, Bio X Cell, Inc.).

For all the experiments, body weight was evaluated and tumor diameters were measured with a caliper, and volumes were calculated with the following formula for ellipsoid volume: 0.5 x D x d^2^, where D is the long and d is the short diameter.

Tumor volumes were compared at each time point using two-way ANOVA followed by Bonferroni’s a posteriori test.

All animals were housed at constant temperature and humidity, according to institutional guide-lines and maintained under standard pathogen-free conditions. The Istituto di Ricerche Farmacologiche Mario Negri IRCCS adheres to national and international laws, regulation and policies on the maintenance, care and use of laboratory animals.

*In vivo sample collection.*

Blood was obtained from the retro-orbital plexus under isoflurane anesthesia and collected in heparinized tubes. The plasma fraction was immediately separated by centrifugation (4,000 rpm, 15 min, 4 C) and stored at −20°C until analysis. After the blood collection mice were sacrifice through dislocation and tumors, livers, muscles and spleens were removed and immediately frozen at −80°C until the molecular analysis.

*Histopathological examination.*

For histopathological examination, the explanted samples were fixed in 10% neutral-buffered formalin for 24 hours and paraffin embedded. Five µm-thick sections obtained from the paraffin blocks were stained with Hematoxylin and Eosin (H&E) and evaluated under a light microscope (Leica DM 2500). Representative images were captured with a digital camera (Leica DFC310 FX). Histopathology evaluation was made in a blind fashion without knowledge of the treatment group.

The histopathological examinations were conducted at the Mouse & Animal Pathology Laboratory of University of Milan.

*ELISA and Multiplex ELISA assays.*

IL-6 was quantified using a high-sensitivity ELISA kit for mouse proteins (Thermo Fisher Scientific #BMS603HS), following the provided instructions. Briefly, sera were added to the coated wells with IL-6 monoclonal antibody in 96-well plates and incubated for 1 hour at room temperature. After four washes, they were incubated with an HRP-linked streptavidin solution for 30 minutes at room temperature in the dark. All samples were tested in duplicate and absorbance was measured at 620 nm using a GloMax Discover plate reader (Promega). Values below the detection limit were excluded from the analyses and related graphs.

Using the MILLIPLEX® MAP Mouse High Sensitivity T Cell Panel kit Merck Life Science (#MHSTCMAG-70K), following the provided instructions we analyzed the levels of the following molecules: interferon gamma (IFNγ), tumor necrosis factor α (TNFα), interleukins (IL) 1β, 5, 12, 13, and 17. Briefly, sera were incubated with the selected antibody-immobilized beads overnight at 4°C in the dark. The next day, after the appropriate washes in each well, the detection antibody was added and incubated for 1 hour at room temperature. At the end of the incubation, a Streptavidin-Phycoerythrin solution was added and incubated for 30 minutes at room temperature. After the appropriate washes in each the Sheath Fluid PLUS was added and the florescence signals were read with BioRad Bio-Plex 200 Paltform.

*Real-Time PCR.*

RNA from the samples were purified using the Simply RNA Maxwell Total RNA Purification Kit (Promega). Total RNA was reverse transcribed to cDNA with the High-Capacity cDNA Retrotranscription Kit (Applied Biosystems) according to the manufacturer’s instructions using Applied Biosystems Veriti 96-well Thermal Cycler (Thermo Fisher Scientific). Primers were purchased for all genes as ready-to-use solutions.

Optimal primer pairs for selected genes (Supplementary Table S1) were designed using PRIMER-3 software (<https://primer3.ut.ee>).

For the quantitative analysis of gene expression, cDNA was amplified by real time RT-PCR (7900HT Fast Real-Time PCR System), with the SYBR Green technique. Relative quantification of mRNA was done using the Δ∆Ct method. Actin was used as internal control. Table 1 reports the sequence of the oligonucleotides used.

**Supplementary Table S1** Sequence of the oligonucleotides used for RT-PCR analysis.

|  | Forward | Reverse |
| --- | --- | --- |
| IL-6 | AGT TCC TCT CTG CAA GAG AC TTC | TCT CCT CTC CGG ACT TGT GAA |
| Atrogin-1 | GTG AGG ACC GGC TAC TGT G | ATC AAA CGC TTG CGA ATC TGC |
| MuRF1 | AAC TGG AGA CCG CCA TC | TGA GCT GCT TGG CAC TTG AG |
| Actin | GGC TGT ATT CCC CTC CAT CG | CCA GTT GGT AAC AAT GCC AT GT |
| UCP1 | GAC AGT ACC CAA GCG TAC CAA | GTC GCA GAA AAG AAG CCA CAA |

*Statistical Analysis.*

Statistical analyses were done with GraphPad Prism 9. The Figure legends include the test used for each experiment. Differences between groups were considered statistically significant when p≤ 0.05.

1. Caiola E, Iezzi A, Tomanelli M, et al. LKB1 Deficiency Renders NSCLC Cells Sensitive to ERK Inhibitors. *J Thorac Oncol*. 2020;15(3):360-370. doi:10.1016/j.jtho.2019.10.009

2. Marabese M, Marchini S, Sabatino MA, et al. Effects of inducible overexpression of DNp73α on cancer cell growth and response to treatment in vitro and in vivo. *Cell Death Differ*. 2005;12(7):805-814. doi:10.1038/sj.cdd.4401622
